# Trends in diagnostic approaches for pediatric appendicitis: nationwide population-based study

**DOI:** 10.1186/s12887-017-0940-7

**Published:** 2017-11-03

**Authors:** Chih-Cheng Luo, Wen-Kuei Chien, Chen-Sheng Huang, Hung-Chieh Lo, Sheng-Mao Wu, Hung-Chang Huang, Ray-Jade Chen, Hsun-Chin Chao

**Affiliations:** 1 Division of Pediatric Surgery, Department of Surgery, Wan Fang Hospital, Taipei City, Taiwan; 2 Department of Surgery, School of Medicine, College of Medicine, Taipei Medical University, Taipei City, Taiwan; 3 Biostatistics Center, Taipei Medical University, Taipei City, Taiwan; 4 Department of Traumatology, Wan Fang Hospital, Taipei City, Taiwan; 5 Department of Acute Care Surgery and Traumatology, Taipei Medical University Hospital, Taipei City, Taiwan; 6 Department of Surgery, Taipei Medical University Hospital, Taipei City, Taiwan; 7 Division of Pediatric Gastroenterology, Department of Pediatrics, Chang Gung Children’s Medical Center, Chang Gung Memorial Hospital, Chang Gung University College of Medicine, 5 Fu-Hsing Street, Guishan Dist, Taoyuan City, 33305 Taiwan

**Keywords:** Appendicitis, Ultrasound, Computed tomography, National Health Insurance Database

## Abstract

**Background:**

To define the benefits of different methods for diagnosis of pediatric appendicitis in Taiwan, a nationwide cohort study was used for analysis.

**Methods:**

We identified 44,529 patients under 18 years old who had been hospitalized with a diagnosis of acute appendicitis between 2003 and 2012. We analyzed the percentages of cases in which ultrasound (US) and/or computed tomography (CT) were performed and non-perforated and perforated appendicitis were diagnosed for each year. Multivariate logistic regression analyses were performed to evaluate risk factors for perforated appendicitis.

**Results:**

There were more cases of non-perforated appendicitis (*N* = 32,491) than perforated appendicitis (*N* = 12,038). The rate of non-perforated cases decreased from 0.068% in 2003 to 0.049% in 2012; perforated cases remained relatively stable at 0.024%~0.023% from 2003 to 2012. The percentage of CT evaluation increased from 3% in 2003 to 20% in 2012; the rates of US or both US and CT evaluations were similar annually. The percentage of neither CT nor US evaluation gradually decreased from 97% in 2003, to 79% in 2012. The odds ratios of a perforated appendix for those patients diagnosed by US, CT, or both US and CT were 1.227 (95% confidence interval (CI) 0.91, 1.65; *p* = 0.173), 2.744 (95% CI 2.55, 2.95; *p* < 0.001), and 5.062 (95% CI = 3.14, 8.17; *p* < 0.001), respectively, compared to patients who did not receive US or CT. The odd ratios of a perforated appendix for those patients 7–12 and ≤6 years old were 1.756 (95% CI 1.67, 1.84; *p* < 0.001) and 3.094 (95% CI 2.87, 3.34; *p* < 0.001), respectively, compared to those 13–18 years old.

**Conclusions:**

Our study demonstrated that using CT scan as a diagnostic tool for acute appendicitis increased annually; most patients especially those ≤6 years old who received CT evaluation had a greater risk of having perforated appendicitis. We recommend a prompt appendectomy in those pediatric patients with typical clinical symptoms and physical findings for non-complicated appendicitis to avoid the risk of appendiceal perforation.

## Background

Appendectomies are one of the most common general surgical procedures performed in the pediatric population. Traditionally, a diagnosis of appendicitis in both children and adults is made by history taking and a physical examination. In general, it is more difficult to obtain a clear history and elicit specific physical examination findings in children of all ages compared to adults [1]. A clinical diagnosis of appendicitis is often difficult, and a delayed diagnosis may result in perforation of the inflamed appendix, peritonitis, or intra-abdominal abscess formation.

Recently, ultrasound (US) and computed tomography (CT) have been used to assist in diagnosing appendicitis. US was initially used [2], but focused CT has become increasingly common as a diagnostic tool in both adults and children to rule out appendicitis in hopes of improving the diagnostic accuracy [3, 4]. Both diagnostic procedures have proven to be much sensitive and specific [5]. Perforation rate of pediatric appendicitis was relatively high in preschool age group and the rate of perforation was inversely proportional to patient age, occurring in 57% ages 4–5 years to 100% aged <1 year [6]. Even with advances in US and CT imaging, perforation rates in children under 6 years was 51–100% over past decades [7–9], therefore there are still critics who question the overlap of these two diagnostic procedures and the benefits of CT over US in terms of the clinical diagnosis [10–12].

As the use of CT and US appears to be increasing, we sought to analyze a large, national database over a 10-year period to evaluate changes in the diagnostic approaches and the impact on the occurrence of perforated appendicitis.

## Methods

### Database

This study was a nationwide, retrospective, population-based analysis of insurance claims data from 23 million insured people obtained from Taiwan’s National Health Insurance (NHI) program. The Bureau of NHI (BNHI) in Taiwan has released a research-oriented database through the Collaboration Center for Health Information Application (CCHIA). Taiwan launched the NHI program in 1995, which covered 99% of the population of Taiwan in 2007. Therefore, the BNHI allows researchers to trace almost all utilizations of medical services for all children with appendicitis in Taiwan.

We used data sourced between 2003 and 2012 from the NHI database released by the BNHI of NHI through the CCHIA. The database includes all original claims data and registration files for beneficiaries enrolled under Taiwan’s NHI program.

This study was exempted from full review by the Taipei Medical University-Joint Institutional Review Board (No: 201,404,074) since the NHI database consists of anonymous secondary data released to the public for research purposes.

### Study sample

We identified 44,529 pediatric patients (< 18 years of age) who had a first-time discharge diagnosis of acute appendicitis (International Classification of Disease, Ninth Revision, Clinical Modification (ICD-9-CM) codes 540.0, 540.1, and 540.9) between January 2003 and December 2012. If a patient had two or more hospitalizations within a 30-day period, they were regarded as the same episode, and we only included the first hospitalization.

we hypothesize that there is possible correlation of age factors in the perforation of pediatric appendicitis, patients were divided into three groups by age: ≤ 6, 7–12, and 13–18 years old. The incidence of disease and the severity of disease were compared among groups. The types of diagnosis were categorized as follows: US (19005B), CT (33070B and 33071B), both US and CT, and neither US nor CT.

We calculated the percentages of cases on which the four categories of diagnostic tools were performed for each year, and also calculated the percentages of non-perforated and perforated appendicitis cases diagnosed each year.

### Outcome measures

1. Primary endpoint measure

Possible correlation of age factors in the perforation of pediatric appendicitis is measured. The initial hypothesis predicts that younger patients are under higher risk of perforation in appendicitis.

(2) Secondary endpoint measure

Both US or CT examinations require scheduling and waiting time; we infer such latency from clinical suspicion to confirmation of diagnosis may increase the risk of appendiceal perforation.

### Statistical analyses

Chi-squared tests were used to examine the difference between the perforated and non-perforated (control) groups. We then performed a multivariate logistic regression to explore the odds ratios (ORs) and the related 95% confidence interval (CI) of perforated appendicitis cases among the different age groups, between genders, and among different diagnostic groups. All statistical analyses were performed using SAS version 9.3 (SAS Institute, Cary, NC), and *p* < 0.05 was considered statistically significant.

## Results

### Characteristic of the study population

Table 1 shows the distributions of rates of acute non-perforated and acute perforated appendicitis cases between genders and among different age groups. Of the 44,529 pediatric patients under 18 years old admitted for treatment of acute appendicitis between January 2003 and December 2012, 26,792 (60%) were boys and 17,737 (40%) were girls. There were more cases of non-perforated appendicitis (*N* = 32,491) than perforated appendicitis (*N* = 12,038).

**Table 1:** Baseline patient characteristics

Non-perforated appendicitis (*N* = 32,491) No %			Perforated appendicitis (*N* = 12,038) No. %		*p-*value*	
Gender						
Male	19,494	60	7298	60.62	0.2346	
Female	12,997	40	4740	39.38		
Age group (y/o)						
≤ 6	2383	7.33	2014	16.73	<0.0001*	
7–12	11,575	35.63	5205	43.24		
13–18	18,533	57.04	4819	40.03		

*Significant differences: *p* < 0.05

Most of the patients (90.1%) were aged between 7 and 18 years. As shown in Table 1, the youngest age group patients (≤ 6 years old) experienced the highest incidence of perforated appendicitis (46%), which decreased to 31% in the 7–12 year age group and then to 21% in the 13–18 year age group.

### Annual incidence of non-perforated appendicitis and perforated appendicitis

**Fig. 1:**
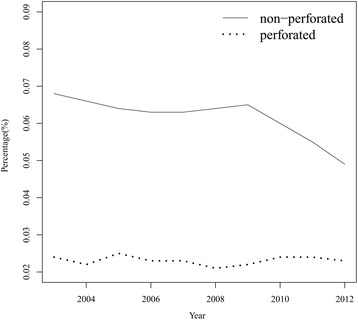
Percentages of patients with non-perforated vs. perforated appendixes from 2003 to 2012

Figure 1 shows that the incidence of the cases of non-perforated appendicitis significantly decreased over the study time course. The incidence of the cases of non-perforated appendicitis were 0.068% in 2003, 0.066% in 2004, and decreased to 0.055% in 2011 and 0.049% in 2012. Interestingly, the incidence of perforated appendicitis cases remained relatively stable at 0.024%~0.023% from 2003 to 2012.

### Annul comparison of the performance of US and CT evaluation

**Fig. 2:**
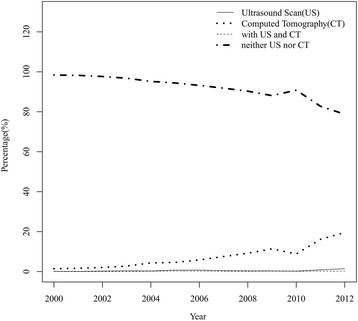
Percentages of patients who received ultrasound (US) and computed tomography (CT)

Figure 2 shows increasing trend in the performance of CT evaluation over the 10-year period. The percentage of CT evaluation increased from 3% in 2003, 4% in 2004, to 20% in 2012, the percentage of US evaluation or combined US with CT evaluation were relatively similar from 2003 to 2012. The percentage of the patients proceeding to an appendectomy without evaluation of US and CT gradually decreased from 97% and 95% in 2003 and 2004, respectively, to 79% in 2012.

### Predictors for appendiceal perforation in appendicitis

Table 2 provides the adjusted ORs for a perforated appendix. Compared to the patients without evaluation of US and CT, the adjusted ORs for a perforated appendix for those patients diagnosed by US, CT, and both US and CT were 1.227 (95% CI 0.91, 1.65; *p* = 0.071), 2.744 (95% CI 2.55, 2.95; *p* < 0.001), and 5.062 (95% CI 3.14, 8.17; *p* < 0.001). Higher rates of perforated appendices were detected among patients between 7 and 12 years old and <6 years old and were 1.756 (95% CI = 1.67, 1.84; *p* < 0.001) and 3.094 (95% CI 2.87, 3.34; *p* < 0.001), respectively, compared to those aged 13–18 years.

**Table 2:** Adjusted odds ratios for perforated appendicitis

Variables	ORs	95% CI	*p-value* *
Gender			
Male	1	**–**	*–*
Female	0.985	0.94–1.03	0.5372
Age group (y/o)			
13–18	1	–	–
7–12	1.756	1.67–1.84	<0.0001*
≤ 6	3.094	2.87–3.34	<0.0001*
Type of diagnosis			
Neither US nor CT	1	–	–
US	1.227	0.91–1.65	0.1729
CT	2.744	2.55–2.95	<0.0001*
Both US and CT	5.062	3.14–8.17	<0.0001*

*ORs* odds ratios, *CI* confidence interval, *US* ultrasound*, CT* computed tomography

*Significant differences: *p* < 0.05

## Discussion

To the best of our knowledge, this is the first study to encompass a large nationwide database to investigate diagnostic approaches for appendicitis in children. The data herein demonstrated that the percentage of children who underwent CT scan increased annually from 2003 on, and still 79% of patients with appendicitis were only diagnosed by clinical judgment. We also found that most patients, especially those aged ≤6 years, who received a CT scan were more likely to have a greater proportion of perforated appendices.

Appendicitis is the most common surgical emergency in children. Our population-based study demonstrated that the largest number of patients was in the 11–18-year age group, which represented 75% of the total population. The ratio of boys to girls was about 1.5:1. A previous report showed an incidence peak in the 10~19-year age group, and it was estimated that the risks of appendicitis were 8.6% for men and 6.7% for women [13]. In our study, the number of cases of non-perforated appendicitis (*n* = 32,491) was higher than that of perforated appendicitis (*n* = 12,038).

Despite great familiarity with this disease, appendicitis continues to pose a significant diagnostic challenge for clinicians [14]. This is especially true in very young children whose history is not typical and whose examination results are also unreliable [15, 16]. It is a trend to use US or CT examination to assist the diagnosis of appendicitis in children in these decades. In our country, majority of the hospital or medical center use US and/or CT to diagnose pediatric appendicitis. In the early 1990s, multiple institutions advocated US as a useful adjunct for diagnosing appendicitis [17–19]. In addition, initial reports by Rao using CT scans to diagnose appendicitis in adults in 1997 led to it being used more frequently in pediatric populations [3, 4]. Both diagnostic procedures have proven to be very sensitive and specific, and appeared to be the preferred imaging modalities for appendicitis in children in our country since 2003. In our series, the use of CT scan significantly increased over the 10-year period. The percentage of the patients who having CT scan increased from 3% to 20%, and the percentage of the patients proceeding to an appendectomy without evaluation of US and CT gradually decreased from 97% to 79%.

Previous studies debated the impacts of CT scans on negative appendectomy and perforation rates [7, 20, 21]. In our series, the incidence of non-perforated appendicitis significantly decreased over the study time course. Interestingly, the rate of perforations remained relatively stable at 0.024%~0.023% from 2003 to 2012. The increased utility of US and CT did not affect the outcome of perforated appendicitis over the study period, but the incidence of non-perforated appendicitis reduced. The reason may be that those patients with suspicious appendicitis without US or CT confirmation may undergo negative appendectomies. If those patients received either or both study, the negative appendectomies may be avoided. The data of ORs demonstrated that patients who received CT or both US and CT had higher rates of the occurrence of perforated appendicitis, especially patients who were ≤6 years old. In our evaluation, the youngest age group (≤ 6 year) had the highest incidence of appendiceal perforation (46%). Various studies have reported ruptured appendicitis rates of 30%~45% [22]. In the study, we are not able to collect the exact data of waiting time for the US or CT examinations in the patients, but both examinations require scheduling and waiting time that should hold true in most circumstances. We infer that the latency from clinical suspicion of acute appendicitis to confirmation of diagnosis by CT or US examinations may increase the risk of appendiceal perforation. A controversial adult report indicated that neither the use of CT nor US led to improve the diagnostic accuracy for acute appendicitis, these procedures might delay surgical consultation and necessary appendectomy. [23] Most hospital institutions have reached a general consensus that for adult patients with suspicion of acute appendicitis, selective use of imaging studies is recommended [24]. The diagnosis or exclusion of appendicitis may be made clinically. However, imaging studies may reduce the negative appendectomy rate [4]. Schuler et al. indicated that the use of CT reduced the negative appendectomy rate for adult patients from 21% without the use of preoperative CT, to 6% with the use of CT [25]. The causal relationship between imaging studies and the occurrence of perforated appendicitis in pediatric acute appendicitis is unknown due to lack of evidence in the literature or research. This is beyond the scope of current study; a future longitudinal study is needed to clarify such relationship.

There are some limitations to this study. The database (the Collaboration Center for Health Information Application) in the study did not provide the socio-economic status, duration of symptoms, and presenting complaint/physical signs. Most medical expenses are covered by national health insurance in our country; therefore socio-economic factor is relatively less relevant in the current study. First, the detailed pathologic confirmation of appendicitis was not available from the database we used, and the definition of appendicitis mainly depended on ICD-9 codes. Second, the dataset in this study had no data on pre-hospital care (the time from the onset of symptoms to first seeking medical attention) of the duration of advanced testing, and the data about the latency from the clinical suspicion of acute appendicitis to the confirmation of diagnosis by US or CT examinations was not available. Therefore, these potentially confounding factors could not be considered in our analysis. Future studies collaborating with other medical centers are expected in order to obtain more detailed clinical data for further analysis. Third, the clinical information about negative appendectomy was not available from the database of the study because negative appendectomy has no suitable ICD-9 to match, which may help to explain the reason for decreased incidence of non-perforated appendectomies in our study, a future longitudinal study is needed to clarify such relationship.

Knowledge of the risks of cumulative radiation exposure from radiographic procedures has led to campaigns aimed at increasing awareness and decreasing radiation exposure [26–30]. Children are more radiosensitive, receive large effective doses for a given level of radiation, and have a longer life expectancy during which to develop cancer [29, 30]. Some recent reports advocated the use of US followed by CT scans [31, 32]. The accuracy of pediatric US in those reports varies from 44% to 94% and the specificity from 47% to 95%. In our series, using US was not popular in the past 10-year period. We suggest that using US instead of CT as the initial modality of diagnosing appendicitis can be done to reduce radiation exposure.

## Conclusions

In summary, our study demonstrated that using CT scan as a diagnostic tool for children’s acute appendicitis gradually increased annually we evaluated. The patients especially those ≤6 years old who received CT evaluation are under greater risk of perforated appendicitis. Further analysis of risk factors for a greater risk of perforated appendicitis in those younger patients who received CT scan is needed. We further emphasize the importance of prompt appendectomy in those pediatric patients with typical clinical symptoms and physical findings for an acute non-complicated appendicitis to avoid the appendiceal perforation and its related medical issue and complications.

## Abbreviations

BNHI: Bureau of NHI
CCHIA: Collaboration Center for Health Information Application
CI: Confidence interval
CT: Computed tomography
ICD-9-CM: International Classification of Disease, Ninth Revision, Clinical Modification
NHI: National health insurance
ODs: Odds ratios
US: Ultrasound
